# Postoperative Sigmoidoscopy and Biopsy After Elective Endovascular and Open Aortic Surgery for Preventing Mortality by Colonic Ischemia (PSB-Aorta-CI): Protocol for a Prospective Study

**DOI:** 10.2196/39071

**Published:** 2022-12-13

**Authors:** Artur Rebelo, Ulrich Ronellenfitsch, Jumber Partsakhashvili, Endres John, Carsten Sekulla, Sebastian Krug, Jonas Rosendahl, Patrick Michl, Jörg Ukkat, Jörg Kleeff

**Affiliations:** 1 Department of Visceral, Vascular and Endocrine Surgery Universitätsklinikum Halle Halle Germany; 2 Department of Gastroenterology Universitätsklinikum Halle Halle Germany

**Keywords:** aortic, colonic, ischemia, surgery, vascular, cardiology, heart disease, patient treatment, clinical decision, health safety, risk assessment

## Abstract

**Background:**

Endovascular aortic repair is considered the standard procedure in treating patients diagnosed with pathologies of the abdominal aorta with suitable anatomy. Open surgery remains an option mostly for patients not suitable for endovascular surgery. Colonic ischemia is an important and life-threatening postoperative complication of these procedures.

**Objective:**

The aim of this study is to evaluate the clinical value and safety of performing a planned sigmoidoscopy and biopsy for detection of colonic ischemia in patients undergoing elective aortic surgery. We also aim to develop prediction scores which could identify patients at risk for colonic ischemia and facilitate their timely treatment.

**Methods:**

The trial is designed as a prospective study. The decision for aortic surgery and eligibility for these procedures will be ascertained according to current guidelines. Afterward, screening of the patient for the remaining inclusion and exclusion criteria will occur. If eligibility for study inclusion is confirmed, the patient will be informed about the aims of the study and all study-specific procedures (sigmoidoscopy and biopsy) and asked to provide informed consent.

**Results:**

The primary end point is the proportion of patients diagnosed endoscopically with subclinical and clinically relevant colonic ischemia among all patients undergoing aortic surgery. Patient recruitment started on June 2021. The final patient is expected to be treated by the end of June 2023. Institutional Review Board review has been completed at the University of Halle (Saale; reference #052-2021).

**Conclusions:**

this shows that sigmoidoscopy can be performed safely and is effective for the timely diagnosis of colonic ischemia in these patients, this could result in its routine implementation in both elective and emergency settings.

**Trial Registration:**

German Clinical Trials Register DRKS00025587; https://www.drks.de/drks_web/navigate.do?navigationId =trial.HTML&TRIAL_ID=DRKS00025587

**International Registered Report Identifier (IRRID):**

DERR1-10.2196/39071

## Introduction

Endovascular aortic repair (EVAR) is considered the standard procedure in treating patients diagnosed with abdominal aortic pathologies and suitable anatomy. Open surgery remains an option mostly for patients not suitable for endovascular surgery. Colonic ischemia is known as a severe complication after both open and endovascular aortic surgery. Its incidence is well described, and even with modern treatment options, colonic ischemia carries a high mortality rate. One of the assumed causes of colonic ischemia after aortic surgery is reduced blood flow via the inferior mesenteric artery. The origin of the vessel is either ligated during open repair (OR) or excluded from antegrade perfusion during EVAR. Reimplantation is rarely performed during OR and not possible during EVAR. Moreover, stenosis or occlusion of the inner iliac arteries may contribute to ischemia of the distal colon and rectum.

**Figure 1:**
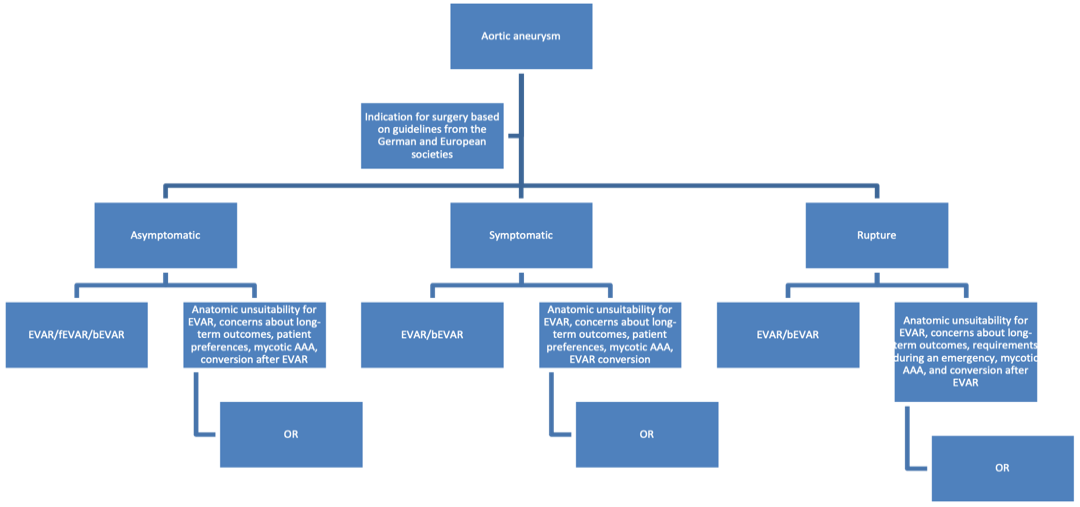
Flowchart of surgical procedures. AAA: adominal aortic aneurysm; bEVAR: branched endovascular aortic repair; fEVAR: fenestrated endovascular aortic repair; OR: open repair.

When compared to OR, EVAR is associated with reduced invasiveness, less general anesthesia, shorter operation times, less blood loss, and lower postoperative pain. Two meta-analyses showed lower short-term mortality in patients undergoing EVAR when compared to OR [1,2]. Nevertheless, there are no advantages in terms of long-term mortality [3]. In the emergency setting, no advantages for EVAR in terms of short- or long-term outcomes could be found [4-7]. Because of the evidence and experience from the last 30 years, most vascular surgeons now consider EVAR as the treatment of choice in the elective and emergency context, leaving OR for individual cases with anatomic unsuitability for EVAR, mycotic abdominal aortic aneurysms, conversion after early or delayed failure or infection of EVAR, or patient preferences (Figure 1).

As already stated, the risk of developing colonic ischemia is present in both endovascular on open treatment of aortic aneurysms. In a meta-analysis involving 13 studies reporting outcomes of colonic ischemia after elective aortic repair comprising 162,750 patients (78,151 EVAR and 84,599 OR), all studies found a higher risk of colonic ischemia for OR than for EVAR (2.1%-3.6% vs 0.5%-1%; combined odds ratio 2.7, 95% CI 2.0-3.5) [8].

Other studies highlight the low incidence but high fatality of colonic ischemia. Lowe et al [9] reported in a systematic review and meta-analysis including 5 cohort studies and 3 case reports with 6184 infrarenal elective EVARs an, incidence of colonic ischemia of 0.5% to 2.8%. In these patients, fatality ranged from 35% to 80%. The authors highlight the importance of identifying risk factors and establishing prophylactic measures in patients with an increased risk of developing these severe complications after infrarenal EVAR. In a database analysis from the United States on 89,967 patients who underwent aortic surgery, the overall incidence of colonic ischemia was 2.2%. The patients who developed colonic ischemia were at increased risk for mortality (37.8% vs 6.7%) [10]. Studies reporting on the use of colonoscopy for identifying patients at risk for colonic ischemia have been published, mostly regarding patients in an emergency context. In both patient collectives—elective and emergent—high mortality rates have been reported. In a study by Champagne et al [11] that included 88 patients who underwent emergent aortic reconstruction because of ruptured aortic aneurysm, colonoscopy was performed in 62 patients who survived for more than 24 hours. Bowel ischemia was documented in 35% of patients. Of these, 16 patients had grade I or grade II ischemia and repeat endoscopy. Grade I ischemia was defined as mucosal ischemia; grade II ischemia was characterized by involvement of the mucosa and the muscularis layers; and grade III ischemia was described by transmural ischemia, gangrene, and perforations. Nine patients needed bowel resection because of grade III ischemia; two procedures were performed because of worsening ischemia discovered at repeat colonoscopy. In another meta-analysis, the accuracy of routine endoscopy in diagnosing colonic ischemia after abdominal aortic aneurysm repair was analyzed. In all, 718 patients were included (44% underwent elective, 56% emergent, and 6% endovascular repair). Among all patients, 20.8% were identified with colonic ischemia (6.5% grade III). The pooled diagnostic odds ratio for all grades of colonic ischemia on endoscopy was 26.6 (95% CI 8.86-79.88) [12]. In a study from Germany, the incidence of colonic ischemia among patients who underwent aortic surgery was 2.4%. Colonic ischemia was predominantly diagnosed by endoscopy (74%). In-hospital mortality was increased in the colonic ischemia group (26.7% vs 2.9%; *P*<.001) [13].

The aim of this study is to evaluate the clinical value and safety of performing a planned sigmoidoscopy and biopsy for detection of colonic ischemia in patients undergoing elective aortic surgery. We also aim to develop prediction scores which could identify patients at risk for colonic ischemia and facilitate their timely treatment. If this study shows that sigmoidoscopy can be performed safely and is effective for diagnosing colonic ischemia in these patients, this could result in its routine implementation in both elective and emergency settings.

## Methods

### Study Design

The trial is designed as a prospective study. The decision for aortic surgery and eligibility for these procedures will be ascertained according to the current guidelines from the German and European societies (Deutsche Gesellschaft für Gefäßchirurgie [DGG; German Society of Vascular Surgery and Vascular Medicine], Society of Vascular Surgery [SVS], and European Society of Vascular Surgery [ESVS]) [14-16]. Afterward, screening of the patient for the remaining inclusion and exclusion criteria will occur. If eligibility for study inclusion is confirmed, the patient will be informed about the aims of the study and all study-specific procedures and asked to provide informed consent. This will take place prior to the planned aortic surgery.

### End Points

The primary end point is the proportion of patients diagnosed endoscopically with subclinical and clinically relevant colonic ischemia among all patients undergoing aortic surgery.

The secondary end points are the proportion of patients requiring surgery for colonic ischemia after aortic surgery, the proportion of patients requiring stoma formation for colonic ischemia, perioperative in-hospital morbidity and mortality measured according to the Clavien-Dindo classification of surgical complications, length of hospital stay, and frequency of reoperation.

### Study Population

The inclusion criteria are the following: elective open or endovascular surgery for abdominal aortic aneurysm (Figure 1), provision of written informed consent prior to any study-specific procedures and willingness to comply with treatment and follow-up, and age ≥18 years.

The exclusion criterion is any serious and/or unstable pre-existing medical, psychiatric, or other condition that could interfere with the patient’s safety, provision of informed consent, or compliance with study procedures.

### Number of Trial Participants

We assume that the rate of colonic ischemia diagnosed by sigmoidoscopy within 48 hours after the operation would be 10% in asymptomatic patients. In the current literature, the incidence of colonic ischemia diagnosed by clinical suspicion only in symptomatic patients is approximately 5%. This rate plus 10% of the 95% of asymptomatic patients results in an overall rate of patients with signs of colonic ischemia of 14.5%. In turn, this results in a 1-armed binomial distribution with 120 cases to be compared to the incidence reported in the literature (5%; α=5%; 1-β [power]=80%) [17].

### Analysis of End Points

Analysis of the primary end point will occur as soon as all patients have undergone aortic surgery and sigmoidoscopy and are discharged from the hospital. The primary end point will be presented as a proportion with a 95% CI.

Secondary end points (proportion of patients proceeding to surgery for colonic ischemia after aortic surgery, perioperative in-hospital morbidity, and mortality measured according to the Clavien-Dindo classification of surgical complications) will also be analyzed once the patient has been discharged from the hospital after the respective procedure. The incidence of complications will be presented as a proportion with a 95% CI stratified by the highest Clavien-Dindo grade of all complications that occurred in each patient.

### Recruitment

Participants will be recruited at the institution carrying out the study. Dedicated screening for trial participation will be performed when open and endovascular aortic surgery for the patient is discussed by the interdisciplinary vascular board. If the patient is selected to undergo open or endovascular surgery, informed consent will be presented to the patient by a physician within 24 hours before surgery.

### Trial Implementation and Enrollment

#### Trial Procedures

All relevant trial procedures are displayed in Table 1. The computed tomography (CT) scan is not a study-specific procedure, as every patient who undergoes open or endovascular aortic surgery will receive a preoperative CT scan as routine care.

**Table 1:** Time and events table.

Required measures	Screening/trial entry	Aortic repair	Sigmoidoscopy 24-48 h after aortic repair
Verification of inclusion and exclusion criteria	✓		
Informed consent	✓		
Computed tomography	✓		
Ascertainment of data as detailed in Evaluation and Follow-up	✓	✓	✓
Ascertainment of primary end point			✓
Follow-up exams at the discretion of the treating physician			
Surgery		✓	

#### Sigmoidoscopy

A standard sigmoidoscopy (from the rectum to the transition of the sigmoid to the descending colon) will be performed. The macroscopically visible ischemia will be graded according to the following scheme: grade I = mucosal ischemia; grade II = involvement of the mucosa and the muscularis layers; and grade III = transmural ischemia, gangrene, and perforations.

A biopsy should be always performed, when possible, in macroscopically suspicious areas. When no macroscopic suspicion is present, a biopsy should be taken in the sigmoid colon. Biopsy should differentiate between normal mucosa, the nongangrenous type of ischemia (mucosal atrophy, edema, hyperemia, mild acute inflammation), and the gangrenous type of ischemia (acute wall necrosis).

### Evaluation and Follow-up

#### Trial Entry

Upon trial entry, the following information will be assessed: date of birth and sex; date of diagnosis; diagnosis; symptoms or clinical history of chronic mesenteric ischemia; in case of aneurysm, the maximal transversal diameter; the length of aneurysm neck; history of cardiovascular diseases; CT findings (patency of abdominal branches: superior mesenteric artery, inferior mesenteric artery and celiac artery, lumbar arteries, hypogastric arteries); Eastern Cooperative Oncology Group (ECOG) performance status; and American Society of Anesthesiologists (ASA) status.

#### Surgery

At the time of surgery, the following information will be assessed: date of surgery; type of procedure; duration of surgery; intraoperative complications according to the Clavien-Dindo-classification [18]; blood results at the time of or directly before the procedure, including C-reactive protein, lactate, leukocytes, glomerular filtration rate, creatinine, and hemoglobin; intraoperative hypotension; intraoperative blood loss; and aortic clamp time.

#### Sigmoidoscopy

At the time of sigmoidoscopy, the following information will be assessed and documented in a protocol: date of sigmoidoscopy; examination time; sigmoidoscopy findings (grade I: mucosal ischemia; grade II: mucosa and the muscularis layers; grade III: transmural ischemia, gangrene, and perforations; if grade III ischemia is detected, no biopsy will be performed); complications of the sigmoidoscopy; blood results, including C-reactive protein, lactate, leukocytes, glomerular filtration rate, creatinine, and hemoglobin; and a checklist to assess the degree of clinical suspicion of colonic ischemia filled out by the surgeon (if the checklist does not indicate a suspicion of colonic ischemia this will be defined as subclinical).

At the time of biopsy result, the following information will be assessed and documented in a protocol: date of result, biopsy findings differentiating ischemia in nongangrenous type (mucosal atrophy, edema, hyperemia, mild acute inflammation), and gangrenous type (acute wall necrosis), and ischemia grade.

Independent observers, blinded to the macroscopical findings of the sigmoidoscopy and the clinical course of the patient, will assess the results of the biopsy.

The tissue will be preserved for immunohistochemistry and RNA analysis of ischemia markers (eg, fatty acid binding protein).

#### Discharge or Death of the Patient

In-hospital mortality and complications will be documented according to the Clavien-Dindo classification. For any operations performed due to a complication, details will be recorded (type of procedure, stoma formation etc).

### Ethics Approval

The study has been approved by the Ethical Committee of the Medical Faculty of the Martin-Luther University Halle-Wittenberg (application #2021-052).

## Results

The primary end point is the proportion of patients diagnosed endoscopically with subclinical and clinically relevant colonic ischemia among all patients undergoing aortic surgery. Patient recruitment started on June 2021. The final patient is expected to be treated by the end of June 2023. For each participant, the duration of the trial will be a treatment phase from recruitment into the trial until hospital discharge. The primary end point will be ascertained at the time of obtaining sigmoidoscopy and biopsy results. The total duration of the trial is expected to be 48 months.

## Discussion

This prospective study will provide insight into the clinical value and safety of performing routine sigmoidoscopy and biopsy for the detection of colonic ischemia in patients undergoing elective aortic surgery and further help determine whether the use of sigmoidoscopy in patients after an aortic surgery is safe. It will be conducted according to the presented protocol. The expected results will support health care professionals and patients with aortic aneurysms undergoing endovascular and open surgery in their decision-making concerning a planned sigmoidoscopy after the procedure. Specifically, we expect the results to provide sufficient data to develop risk scores for these patients. If this study shows that sigmoidoscopy can be performed safely and is effective in diagnosing colonic ischemia in these patients, it could be implemented routinely in both elective and emergency settings.

After due consideration, all participating investigators are convinced that the trial has a favorable risk-benefit ratio. The study has been approved by the Ethical Committee of the Medical Faculty of the Martin-Luther University Halle-Wittenberg. The innovative prospective design of this study in this context is also relevant for providing new knowledge in an international research context because, so far, only retrospective studies have been conducted on the given research topic. Colonic ischemia after aortic surgery is a relatively rare condition, with an incidence of approximately 5%. However, it bears severe clinical consequences with an in-hospital mortality of 26.7%-37.8% [9,10,13]. Colonoscopy and sigmoidoscopy have been proven to be sensitive and specific in diagnosing this pathology in the aortic surgery scenario [10-12] and are already a diagnostic standard when there is clinical suspicion [19]. Furthermore, based on the current data, no precise and evidence-based clinical, laboratory, or radiographic instruments that recognize colonic ischemia are available. Sigmoidoscopy has been described as a safe procedure with complication rates of 0.08%, with most complications being of mild severity [20]. However, these complication rates relate to a general population and not to patients after aortic surgery with possible colonic ischemia, in whom the risk for endoscopy-related complications is unclear and might be higher. Nonetheless, the additional risk posed to trial participants by sigmoidoscopy is estimated to be low according to the available cohort studies [20].

We state that according to the currently available evidence, the value of sigmoidoscopy and biopsy after endovascular and open surgery should be investigated to develop prediction scores that could identify patients at risk for colonic ischemia and facilitate their timely treatment. Patients with a delayed diagnosis of the condition have a high mortality. If a patient is diagnosed with subclinical ischemia, follow-up will be offered. All complications, both arising from sigmoidoscopy and the other aspects of the patients’ treatment, will be documented and reported to the principal investigator (AR). He can interrupt the trial at any point or, in accordance with the ethical committee, implement changes to the study protocol.

The study is being conducted in accordance with the applicable version of the declaration of Helsinki. Prior to study initiation, approval from the relevant ethical committee has been sought. Before enrolment into the trial, patients will be informed in writing and verbally about the nature and implications of the trial and especially about the possible benefits and risks to their health. Patients will document their consent by signing the informed consent form. Patients can leave the trial at any point without providing a reason for doing so. In such a case, treatment of the patient will continue according to the individual judgment of the treating physicians. Given that open aortic surgery and sigmoidoscopy are considered routine surgical and diagnostic treatments and given that the trial evaluates the value of sigmoidoscopy as a novel diagnostic sequence, there is no requirement for trial-specific patient insurance. Trial participants will be insured by the hospital’s insurance covering inpatient treatments. The trial has been registered in a publicly available repository (German Clinical Trials Register; DRKS00025587) for clinical trials prior to initiation. All planned substantial changes will be submitted for approval to the relevant ethical committees in writing as protocol amendments.

Concerning our dissemination plan, we aim to publish results from this trial in the form of one or several manuscripts in international medical journals. The principal investigator (AR) will review all manuscripts to prevent forfeiture of patent rights to data not in the public domain. The authorship list will be agreed on by the principal investigator prior to publication. Investigators from the participating departments will be offered authorship on manuscripts. Publication of the first manuscript reporting study results is planned to take place as soon as possible after analysis of the primary end point. Efforts will be made to ensure that the pertinent manuscript will not be submitted later than 6 months after the results are available.

## Abbreviations

ASA: American Society of Anesthesiologists
CT: computed tomography
DGG: Deutsche Gesellschaft für Gefäßchirurgie
ECOG: Eastern Cooperative Oncology Group
ESVS: European Society of Vascular Surgery
EVAR: endovascular aortic repair
OR: open repair
SVS: Society of Vascular Surgery

## Data Availability

The data sets generated during this study will be available from the corresponding author (AR) on reasonable request.
